# Efficacy of Mesoglycan in Pain Control after Excisional Hemorrhoidectomy: A Pilot Comparative Prospective Multicenter Study

**DOI:** 10.1155/2018/6423895

**Published:** 2018-03-19

**Authors:** Gaetano Gallo, Massimiliano Mistrangelo, Roberto Passera, Valentina Testa, Mauro Pozzo, Roberto Perinotti, Ivan Lanati, Ivano Lazzari, Paolo Tonello, Elettra Ugliono, Emilia De Luca, Alberto Realis Luc, Giuseppe Clerico, Mario Trompetto

**Affiliations:** ^1^Department of Colorectal Surgery, Santa Rita Clinic, Vercelli, Italy; ^2^Department of Surgical and Medical Sciences, University “Magna Graecia” of Catanzaro, Catanzaro, Italy; ^3^Department of Surgical Science, Città della Salute e della Scienza Hospital, University of Turin, Turin, Italy; ^4^Nuclear Medicine Department, Città della Salute e della Scienza Hospital, University of Turin, Turin, Italy; ^5^Colorectal Surgical Unit, Department of Surgery, Infermi Hospital, Biella, Italy; ^6^Department of Surgery, Savigliano Hospital, Savigliano, Italy; ^7^Department of Surgery, Koelliker Hospital, Turin, Italy

## Abstract

**Introduction:**

Various pain management strategies for patients undergoing open excisional hemorrhoidectomy have been proposed, yet postoperative pain remains a frequent complaint.

**Objective:**

To determine whether mesoglycan (30 mg two vials i.m. once/day for the first 5 days postoperative, followed by 50 mg 1 oral tablet twice/day for 30 days) would reduce the edema of the mucocutaneous bridges and thus improve postoperative pain symptoms.

**Patients and Methods:**

For this prospective observational multicenter study, 101 patients undergoing excisional diathermy hemorrhoidectomy for III-IV degree hemorrhoidal disease were enrolled at 5 colorectal referral centers. Patients were assigned to receive either mesoglycan (study group SG) or a recommended oral dose of ketorolac tromethamine of 10 mg every 4–6 hours, not exceeding 40 mg per day and not exceeding 5 postoperative days according to the indications for short-term management of moderate/severe acute postoperative pain, plus stool softeners (control group CG).

**Results:**

Postoperative thrombosis (SG 1/48 versus CG 5/45) (*p* < 0.001) and pain after rectal examination (*p* < 0.001) were significantly reduced at 7–10 days after surgery in the mesoglycan-treated group, permitting a faster return to work (*p* < 0.001); however, in the same group, the incidence of postoperative bleeding, considered relevant when needing a readmission or an unexpected outpatient visit, was higher, possibly owing to the drug's antithrombotic properties.

**Conclusions:**

The administration of mesoglycan after an open diathermy excisional hemorrhoidectomy can reduce postoperative thrombosis and pain at 7–10 days after surgery, permitting a faster return to normal activities.

## 1. Introduction

Hemorrhoids are a common, multifactorial benign but bothersome condition [1]. Among the many surgical procedures for their treatment, the ideal one should be minimally invasive, painless, safe, and effective with minimal costs [2–5]. Patients with grade III-IV hemorrhoidal prolapses or those who have failed conservative and outpatient treatments are considered eligible for excisional hemorrhoidectomy [6].

Open excisional hemorrhoidectomy remains the gold standard technique despite the risk of postoperative bleeding and associated sequelae such as anal stenosis and fecal incontinence. Its main drawback is the frequent severe postoperative pain caused by postoperative scars, hygiene/social habits, hard stools, and diet, particularly by the edema of the mucocutaneous bridges, especially if a local thrombosis develops [7–12]. Strategies to reduce postoperative pain rely on medical therapy (metronidazole, diltiazem, glyceryl trinitrate, flavonoids, cholestyramine ointment, liposome bupivacaine, 0.2% glyceryl trinitrate, lignocaine ointments, and atorvastatine) [13–21] as well as on innovative energy-based surgical devices [22–24].

The aim of this prospective comparative observational multicenter study was to determine whether the administration of mesoglycan in patients undergoing hemorrhoidectomy would reduce postoperative pain.

Mesoglycan belongs to a broad class of compounds called glycosaminoglycans that are amino hexose polysaccharides contained in mucoproteins, glycoproteins, and blood group substances. It is composed of dermatan sulfate, heparan sulfate, electrophoretically slow-moving heparin, and minimal quantities of chondroitin sulfate, hyaluronic acid, and related hexosaminoglycans [25]. Mesoglycan exerts its profibrinolytic activity without altering hemagglutination in humans after oral administration and decreases plasma fibrinogen concentrations without affecting prothrombin time, partial thromboplastin time, or antithrombin III. Moreover, it seems to reduce pericapillary connective tissue edema and capillary and venule dilation in patients with primary venous insufficiency. It can also improve arterial wall elasticity, transcutaneous oxygen perfusion, and blood flow [25–30].

Our hypothesis was that for the anti-inflammatory and profibrinolytic activities of the mesoglycan, its use could improve the postoperative pain due to edema and thrombosis of the mucocutaneous bridges.

## 2. Materials and Methods

The study was approved by the local ethical committee of each site, and written informed consent was obtained from all patients.

One hundred and one consecutive patients submitted to open diathermy excisional hemorrhoidectomy for grade III-IV hemorrhoids were enrolled at five colorectal referral centers from January 2016 to April 2016. The exclusion criteria were age < 18 or >80 years; history of coagulopathy, cardiac diseases, anticoagulant therapies, colorectal or anal neoplasms and/or inflammatory bowel disease (IBD) and/or other proctological diseases (anal fistulas and fissures), and pelvic radiotherapy; previous anal surgical procedures; inability to return for postoperative control visits; and known allergy to mesoglycan. Proctological examination and proctoscopy were performed to grade hemorrhoid disease and to exclude any associated proctological or rectal pathologies. Patients aged more than 50 years or with symptoms suggestive of IBD underwent a preoperative pancolonoscopy.

Patients were, alternatively, assigned to receive mesoglycan (Prisma® 30 mg 2 vials i.m./day for the first 5 postoperative days and then Prisma 50 mg 1 oral tablet twice/day for further 30 days, Mediolanum Farmaceutici, Milan, Italy) or standard postoperative therapy, as established before the beginning of the study (a recommended oral dose of ketorolac tromethamine of 10 mg every 4–6 hours, not exceeding 40 mg per day and not exceeding 5 postoperative days according to the indications for the short-term management of moderate/severe acute postoperative pain, plus stool softeners). Mesoglycan i.m. was always administered by a nurse on postoperative day 1 and then for the next 4 days by the same home-care nurse.

All patients underwent an open diathermy excisional hemorrhoidectomy, under spinal or general anesthesia, with removal of the three classical piles. At each of the five centers, the procedure was carried out by a surgeon with at least 200 previous hemorrhoidectomies. Three v-shaped incisions were placed on the mucocutaneous border, leaving a skin bridge in between to avoid anal stenosis. No ligation of the vascular pedicle was applied and no contemporary internal sphincterotomy was performed. Discharge was planned the day after surgery.

Patients were followed up by the same surgeon who had performed the procedure and were scheduled for a control visit at the first postoperative day (T1), at 7 to 10 days (T2), 20 days (T3), and 40 days after discharge (T4). The follow-up assessment comprised clinical external evaluation at T1, clinical evaluation with rectal digital exploration at T2, and clinical evaluation and proctoscopy at T3 and T4. Postoperative pain was evaluated using a visual analog scale (VAS) for pain in which 0 indicates no pain and 10 the worst possible pain.

The presence of thrombosis, defined as one or more swollen painful piles at the site of mucocutaneous bridges, was assessed at T2, T3, and T4.

A postoperative bleeding was considered relevant when needing a readmission or an unexpected outpatient visit.

We considered autonomy as the return to normal activity: it includes also the return to work apart from cases of retired patients in which we considered it as the complete return to daily activities.

The following data were collected: the name of the study site/surgeon; VAS at rest (VAS-sp), VAS after defecation (VAS-def), and VAS after rectal examination (VAS-espl); complications (hemorrhage, edema, and thrombosis); evaluation of surgical scars (granulation and time to healing); and possible autonomy and time of return to work.

All procedures performed in studies involving human participants were in accordance with the ethical standards of the institutional and/or national research committee and with the 1964 Helsinki Declaration and its later amendments or comparable ethical standards. This article does not contain any studies with animals performed by any of the authors.

### 2.1. Statistical Analysis

Due to the explorative, nonconfirmative nature of this research, no formal sample size determination was performed.

Patient characteristics were analyzed using Fisher's exact test for categorical variables and either Mann–Whitney and Kruskal-Wallis tests (for independent measures) or Wilcoxon and Friedman tests (for repeated measures) for continuous variables. Descriptive results for continuous variables are expressed as the median (interquartile range [IQR]). For the continuous variables (VAS-sp, VAS-def, and VAS-espl), the primary outcomes were their independent trends over time and the potential effects of mesoglycan therapy. These three variables were longitudinally measured at the four time points and then used as dependent variables in three independent univariate and multivariate mixed-effects linear model (MLM) series. Since the distributions of the VAS-sp, VAS-def, and VAS-espl time series by single time points were Gaussian, no log-transformation was needed. The three multivariate mixed-effects linear models for the repeated measures of VAS-sp, VAS-def, and VAS-espl were estimated by the restricted maximum likelihood method, using a first-order autoregressive covariance matrix: for every covariate, the variances at each time point were considered comparable and constant, while the correlations between subsequent measures were considered similar.

For the four categorical variables (bleeding, thrombosis, time to healing, autonomy, and return to work), the primary outcomes were their independent trends over time and the potential effects of mesoglycan therapy. The frequencies of these covariates are reported at the same four time points. The changes noted at the follow-up examination and the effects of mesoglycan were evaluated by the generalized estimating equations model (GEE for repeated measures logistic regression). GEE extends GLM (generalized linear model) to longitudinal data, with correlated outcomes; the first-order autoregressive correlation matrix was used to represent the within-subject dependencies. Their trends over time (outcomes and dependent categorical variables) were estimated in four independent GEE models, analyzing the follow-up time after surgery (independent continuous variable) and the effects of mesoglycan (mesoglycan versus control and independent categorical variable) as its determinant. All reported *p*-values were obtained by the two-sided exact method at the conventional 5% significance level. Data were analyzed as of December 2016 using R version 3.3.2 (R Foundation for Statistical Computing, Vienna, Austria, http://www.R-project.org).

## 3. Results

A total of 101 patients with III-IV degree hemorrhoids undergoing open excisional hemorrhoidectomy were assigned to receive mesoglycan (*n* = 54, 21 women and 33 men) or standard postoperative therapy (*n* = 47, 17 women and 30 men). The two groups were similar for sex, age, and grade of disease (Table 1). No intraoperative complications occurred. All patients were discharged on postoperative day 1. The adherence rate was 100%, with good compliance to mesoglycan therapy. No drug-related side effects occurred, and no patient complained about any discomfort depending on i.m. or prolonged oral treatment with mesoglycan.

**Figure 1:**
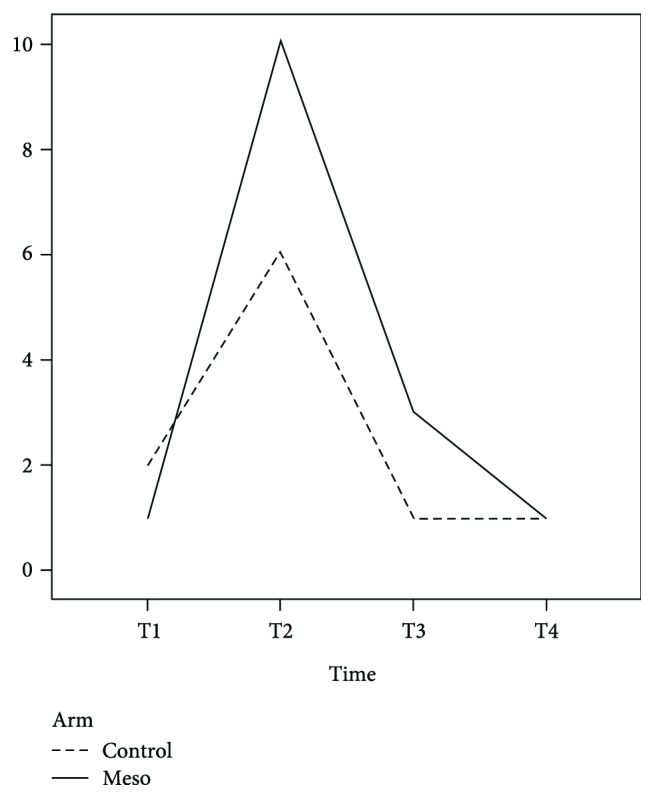
Incidence of postoperative bleeding. The dashed line indicates the control group, and the solid line indicates the mesoglycan-treated group.

**Figure 2:**
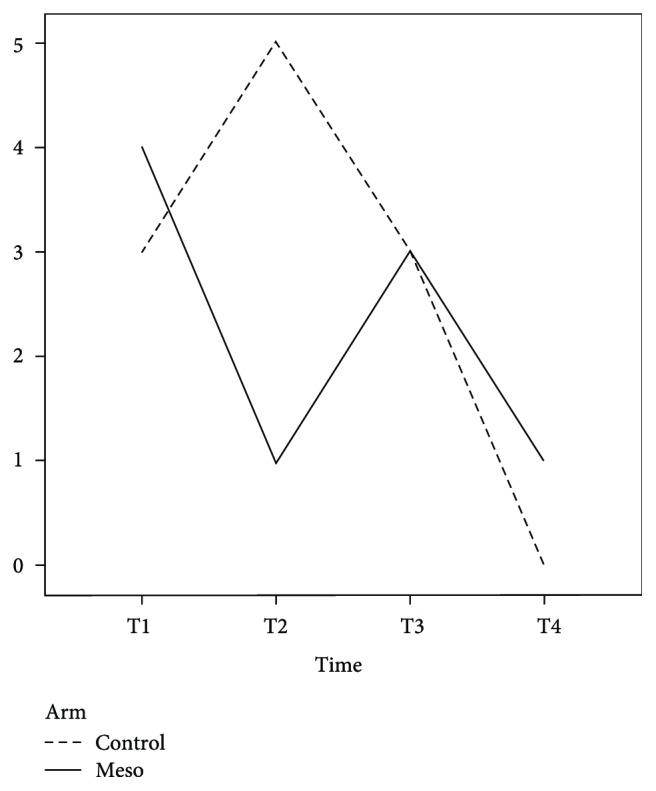
Incidence of postoperative thrombosis. The dashed line indicates the control group, and the solid line indicates the mesoglycan-treated group.

**Figure 3:**
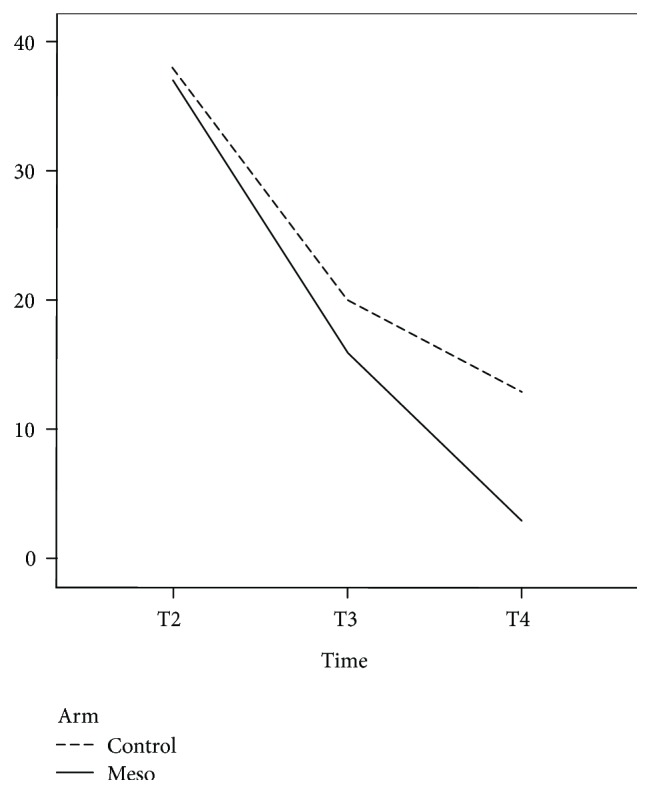
Time to return to work/daily activities. The dashed line indicates the control group, and the solid line indicates the mesoglycan-treated group.

Table 2 presents the outcome data for each group. Pain during defecation was not evaluated at T1 because few patients had bowel movement at this time point, and as expected, mesoglycan therapy did not influence this parameter. The main adverse effect in the mesoglycan-treated group was a higher postoperative bleeding rate at T2, probably secondary to the drug's antithrombotic properties, albeit without statistical significance (*p* = 0.488) (Figure 1). No patients needed further surgery for bleeding. Analysis of the time trend for thrombosis (Table 3) was similar in the two groups (*p* = 0.332), except at T2, when a significant reduction in postoperative thrombosis was observed in the mesoglycan-treated group (*p* < 0.001) (Figure 2). As expected, mesoglycan therapy had no effect on the time to surgical wound healing (*p* = 0.457). The time to return to work or normal daily activities was significantly shorter (*p* = 0.009) in the mesoglycan-treated group (Figure 3).

**Figure 4:**
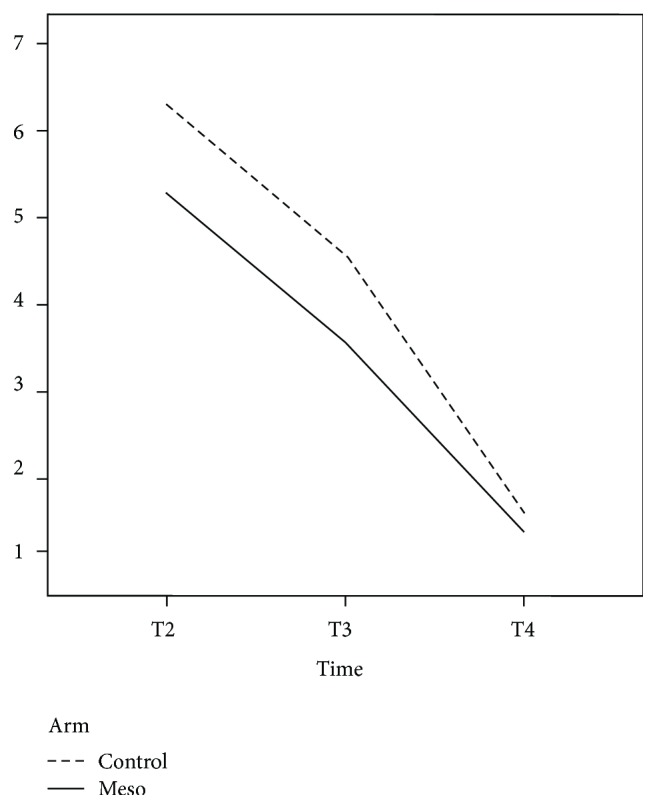
Visual analog pain scale scores after rectal examination. The dashed line indicates the control group, and the solid line indicates the mesoglycan-treated group.

Table 4 presents the effects of mesoglycan therapy on postoperative pain (spontaneous, after defecation, and after rectal examination) at each time point with a statistically significant reduction in pain after rectal examination (*p* = 0.033) for the mesoglycan-treated group (Figure 4). Also, the reduction in postoperative thrombosis, accompanied by improvement in postoperative pain symptoms, permitted a faster return to work.

## 4. Discussion

Postoperative pain is the most common complaint after an excisional hemorrhoidectomy, and it can be the reason for prolonged hospital stay and time off work [5, 6]. While hemorrhoidopexy (Longo's procedure) has demonstrated a clear reduction in postoperative pain as compared with the Milligan-Morgan procedure [31], its use has declined owing to the high percentage of recurrences and the risk of possible severe and sometimes life-threatening complications. These adverse effects have revived interest in the traditional open excisional hemorrhoidectomy, which has become the mainstay of the standard surgical therapy for symptomatic hemorrhoids [32].

Postoperative pain after excisional hemorrhoidectomy generally results from a spasm of the internal anal sphincter and/or from a secondary infection of the surgical site, and many attempts have been proposed to reduce these possible etiopathogenetic factors. Metronidazole has been successfully used since 1998 for treating internal anal sphincter spasm [13], and its benefit after hemorrhoidectomy has been confirmed in a recent study [14]. Similarly, 0.2% glyceryl trinitrate has been successfully used to reduce both anal internal sphincter spasm and postoperative pain [14, 15, 33–36]. Patti et al. reported the effectiveness of both the topical application of 0.2% glyceryl trinitrate ointment and botulinum injection over placebo in reducing postoperative pain at resting or during defecation and improving wound healing [37, 38]. A systematic review evaluating 207 randomized studies, of which 65 were included, reported a significant decrease of postoperative pain with the use of perianal local anaesthetic infiltration [8]. Flavonoids, with their phlebotropic activity, protective effect against the capillary fragility, and anti-inflammatory effect, are the only pharmacological treatment which can be compared to mesoglycan. Filingeri et al. described the use of flavonoids after hemorrhoidectomy with radiofrequency scalpel, demonstrating a reduction of the inflammatory process and edema. In their randomized study, involving 26 patients, the flavonoid group compared to a control group gave no advantage regarding pain and bleeding at 7, 15, and 30 days postop [19].

La Torre and Nicolai demonstrated a significant advantage in terms of postoperative comfort and reduction of pain and bleeding using a combination of micronized purified flavonoid fraction plus short-term antibiotic and anti-inflammatory treatment compared with an antibiotic and anti-inflammatory treatment alone [39].

Owing to its analgesic and anti-inflammatory activities, atorvastatin can reduce the inflammation and irritation of the perianal skin [15], as seen also after the topical application of vitamin E ointment [40].

The aim of the present study was to determine whether the use of a product containing mesoglycan could reduce the local thrombosis and edema of the mucocutaneous bridges after an open excisional hemorrhoidectomy, with a consequent improvement of the postoperative pain.

To our knowledge, this is the first report on the use of mesoglycan in the postoperative treatment of hemorrhoids. Only two old studies reported the use of mesoglycan in patients with acute hemorrhoidal disease [41, 42].

The severe standardization of the pre-, intra- and postoperative managements of the patients is the main strength of our prospective comparative observational multicenter study, limiting the bias of the nonrandomization of the study.

Our results indicate that mesoglycan therapy can reduce postoperative thrombosis and edema after an open diathermy excisional hemorrhoidectomy, with decreased postoperative pain symptoms, statistically significant at T2 after rectal examination. Even though this is the only significant result, we consider the possibility to return to work after 7–10 days after surgery as the main benefit of the mesoglycan from a socio-economical point of view, with a real faster return to work.

In our opinion, these advantages depend on the initial postoperative intramuscular administration of a high dose of the drug. Of note, we also found a trend for a higher rate of postoperative bleeding at T2, probably due the well-known antithrombotic properties of the mesoglycan.

A further interesting result was that mesoglycan was not inferior to standard treatment in controlling postoperative pain at any T.

This study has several limitations. The number of patients is small and may not have sufficient power to reveal small difference in outcomes, but the major methodological flaw is the lack of a uniformed and standardized thrombosis-measuring tool. The different number of patients in the two groups depends on the strength of recruitment of the single centers.

## 5. Conclusion

In our small series of patients, mesoglycan has been useful in reducing the incidence of thrombosis and the severity of pain at digital rectal examination after an open diathermy excisional hemorrhoidectomy at 7–10 days postop.

This has allowed a faster return to normal daily activities. Further studies on larger patient samples are needed to confirm our results.

## Figures and Tables

**Table 1:** Demographic and clinical characteristics.

Characteristic	All patients (*N* = 101)	Mesoglycan-treated group (*N* = 54)	Control group (*N* = 47)
No. of men	63	33	30
Median age (yr)	53 (30–78)	52 (30–78)	53 (34–77)
No. of patients with			
(i) III degree hemorrhoids (ii) IV degree hemorrhoids	85 16	45 9	40 7

**Table 2:** Proportion of patients experiencing pain after defecation, bleeding, absence of thrombosis, time to wound healing, and return to normal activity at each time point (in all patients and in the mesoglycan-treated group) and results of the generalized estimating equations (GEE) models.

	All patients	Meso group	Control group	Fisher's test *p*
No pain at defecation—T2	69/95 (72.6%)	37/50 (74.0%)	32/45 (71.1%)	0.8198
No pain at defecation—T3	76/93 (81.7%)	38/49 (77.6%)	38/44 (86.4%)	0.2974
No pain at defecation—T4	85/95 (89.5%)	45/51 (88.2%)	40/44 (90.9%)	0.7475
No bleeding—T1	94/97 (96.9%)	51/52 (98.1%)	43/45 (95.6%)	0.5953
No bleeding—T2	78/94 (83.0%)	39/49 (79.6%)	39/45 (86.7%)	0.4192
No bleeding—T3	90/94 (95.7%)	47/50 (94.0%)	43/44 (97.7%)	0.6200
No bleeding—T4	91/93 (97.8%)	50/51 (98.0%)	41/42 (97.6%)	1.0000
Arm (meso versus control)				0.488
No thrombosis—T1	90/97 (92.8%)	48/52 (92.3%)	42/45 (93.3%)	1.0000
No thrombosis—T2	87/93 (93.5%)	47/48 (97.9%)	40/45 (88.9%)	0.1037
No thrombosis—T3	88/94 (93.6%)	47/50 (94.0%)	41/44 (93.2%)	1.000
No thrombosis—T4	91/92 (98.9%)	48/49 (98.0%)	43/43 (100%)	1.000
Arm (meso versus control)				0.332
Wound healing—T2	0/93 (0%)	0/48 (0%)	0/45 (0%)	1.000
Wound healing—T3	1/94 (1.1%)	1/50 (2.0%)	0/44 (0%)	1.000
Wound healing—T4	67/93 (72.0%)	38/51 (74.5%)	29/42 (69.0%)	0.6447
Arm (meso versus control)				0.457
Return to activity—T2	16/91 (17.6%)	10/47 (21.3%)	6/44 (13.6%)	0.4144
Return to activity—T3	58/94 (61.7%)	34/50 (68.0%)	24/44 (54.5%)	0.2067
Return to activity—T4	77/93 (82.8%)	46/49 (93.9%)	31/44 (70.5%)	0.0048
Arm (meso versus control)				0.009

**Table 3:** *N* of patients with postoperative thrombosis.

	Control group	Meso group
T1	3/45 (6.7%)	4/52 (7.7%)
T2	5/45 (11.1%)	1/48 (2.1%)
T3	3/44 (6.8%)	3/50 (6.0%)
T4	0/43	1/49 (2.0%)

**Table 4:** Median (IQR) VAS scores for spontaneous pain, pain after defecation, and after rectal examination at each time point and results of the mixed-effects linear (MLM) models.

Variable	All patients	Mesoglycan group	Control group	*p*-value
Spontaneous pain—T1	3 (2–5)	3 (2–4)	4 (3–5)	
(i) T2 (ii) T3 (iii) T4	3 (2–4) 2 (0–3) 0 (0–1)	3 (1–4) 1 (0–3) 0 (0–1)	3 (2–4) 2 (2–3) 0 (0–1)	
Arm (mesoglycan versus control)				0.122
Pain after defecation—T2	6 (5–7)	6 (4–7)	6 (5–8)	
(i) T3 (ii) T4	4 (3–5) 1 (0–2)	3 (2–5) 1 (0–2)	4 (3–5) 2 (1–3)	
Arm (mesoglycan versus control)				0.138
Pain after rectal examination—T2	6 (4–8)	5 (3–7)	6 (5–8)	
(i) T3 (ii) T4	4 (3–5) 1 (0–2)	4 (2–5) 1 (0–2)	5 (3–6) 2 (0–3)	
Arm (mesoglycan versus control)				0.033
